# Steroid Hormone Receptor Signals as Prognosticators for Urothelial Tumor

**DOI:** 10.1155/2015/840640

**Published:** 2015-12-07

**Authors:** Hiroki Ide, Hiroshi Miyamoto

**Affiliations:** Departments of Pathology and Urology, Johns Hopkins University School of Medicine, Baltimore, MD 21287, USA

## Abstract

There is a substantial amount of preclinical or clinical evidence suggesting that steroid hormone receptor-mediated signals play a critical role in urothelial tumorigenesis and tumor progression. These receptors include androgen receptor, estrogen receptors, glucocorticoid receptor, progesterone receptor, vitamin D receptor, retinoid receptors, peroxisome proliferator-activated receptors, and others including orphan receptors. In particular, studies using urothelial cancer tissue specimens have demonstrated that elevated or reduced expression of these receptors as well as alterations of their upstream or downstream pathways correlates with patient outcomes. This review summarizes and discusses available data suggesting that steroid hormone receptors and related signals serve as biomarkers for urothelial carcinoma and are able to predict tumor recurrence or progression.

## 1. Introduction

Bladder cancer, which is mostly urothelial carcinoma, is one of the most frequently diagnosed neoplasms, with estimated 429,800 new cases and 165,100 deaths which occurred in 2012 worldwide [1]. Patients with superficial urothelial tumor suffer from its recurrence with occasional progression to muscle invasion after transurethral surgery. In contrast, those with muscle-invasive tumor often develop disease progression or metastatic tumor despite more aggressive treatment. Cystoscopy which is an invasive and relatively expensive procedure is the “gold standard” for the detection of bladder cancer [2–4]. Urine cytology is a highly specific, noninvasive adjuvant test widely utilized with cystoscopy for both screening/initial diagnosis of bladder cancer and surveillance of tumor recurrence [5]. There are also several urine-based markers/tests, such as nuclear matrix protein 22 (NMP22), bladder tumor antigen (BTA), and UroVysion, which are useful for detecting urothelial tumors and may thus be substitutes of cystoscopy and/or cytology [6–9]. However, none of these markers or tests have demonstrated a significant association with prospective tumor recurrence or disease progression in patients with urothelial cancer.

Epidemiological and clinical studies have indicated that men have a significantly higher risk of bladder cancer, whereas women tend to have more aggressive tumors [1, 10–15]. These observations have prompted investigations of steroid hormones and their receptor signals, especially androgens/estrogens and androgen/estrogen receptors (AR/ER), in bladder cancer, which have demonstrated their critical roles in tumorigenesis and tumor progression [16–18]. Accordingly, bladder cancer is now considered as an endocrine-related neoplasm. Additionally, studies have identified a variety of molecules or pathways regulated by steroid hormones and their receptor signals in bladder cancer cells. These findings have also provided novel therapeutic targets for urothelial carcinoma.

Recent evidence has thus indicated the involvement of nuclear receptor-mediated signals in urothelial cancer outgrowth. These receptors include AR, ER*α*, ER*β*, glucocorticoid receptor (GR), progesterone receptor (PR), vitamin D receptor (VDR), retinoid receptors (e.g., retinoic acid receptor (RAR) and retinoid X receptor (RXR)), and peroxisome proliferator-activated receptors (e.g., PPAR*γ*) as well as orphan receptors. More importantly, recent studies have assessed the prognostic significance of steroid hormone receptor signals and related pathways in urothelial tumors. In this paper, we mainly review immunohistochemical studies showing associations between alterations of steroid hormone receptors in urothelial tumors and patient outcomes. Furthermore, we highlight several molecules regulated by AR and/or ER signals in bladder cancer cells, which may contribute to the development of diagnostic and/or prognostic biomarkers.

## 2. Androgens and AR

Using cell line and animal models, androgens have been shown to promote urothelial carcinogenesis and cancer progression via the AR pathway [16, 17, 19–26]. Specifically, androgen deprivation inhibited tumor development in male rodents treated with a bladder carcinogen* N*-butyl-*N*-4-hydroxybutyl nitrosamine (BBN) [21, 23]. Furthermore, BBN completely failed to induce bladder cancer in AR knockout mice [23]. AR signals have also been found to downregulate the expression of P450 CYP4B1 [27], UDP-glucuronosyltransferases (UGTs) [28], and GATA3 [29], all of which are known to prevent urothelial tumorigenesis. Meanwhile, androgen deprivation resulted in inhibition of bladder cancer cell proliferation and invasion [23–26, 30–33]. Recent clinical studies have also suggested that androgen deprivation therapy for prostate cancer prevents bladder cancer development [34] and recurrence [35].

Immunohistochemical studies have demonstrated that the positive rates of AR expression in bladder or upper urinary tract (UUT) urothelial tumors range from 13% to 55%, which is significantly lower than that in nonneoplastic urothelial tissues [25, 30, 36–46] (Table 1). However, two studies showed no AR expression in normal urothelium [30, 43]. Similarly, most of the studies showed downregulation of AR expression in high-grade and muscle-invasive tumors, compared with low-grade and non-muscle-invasive tumors, respectively [25, 30, 36, 39–41, 45].

Prognostic significance of AR expression in urothelial tumors remains controversial. Despite the promoting effects of AR signals on tumorigenesis, two studies showed a significant correlation [44] and a tendency [30], respectively, between AR expression and lower risks of bladder tumor recurrence. In contrast, AR expression correlated with the progression of bladder tumors [40, 43], while others did not reveal its prognostic significance in patients with bladder cancer. Additionally, none of the immunohistochemical analyses in UUT tumors have demonstrated strong correlations of AR expression with their outcomes.

## 3. Estrogens and ERs

Both stimulatory and inhibitory effects of estrogens on urothelial cancer outgrowth, which appear to be cell-specific and dependent on the functional activity of ER*α* and ER*β*, have been documented [16, 18–20, 47–50]. For instance, significantly higher incidence of bladder cancer was observed in BBN-treated ER*α* knockout female mice, compared with wild-type female littermates, suggesting the preventive role of ER*α* in bladder cancer development [51]. Selective ER modulators, such as tamoxifen and raloxifen, were also shown to inhibit the growth of bladder cancer cell lines expressing ER*β* [47, 49]. Nonetheless, estrogens promoted the cell proliferation of a urothelial cancer line predominantly via the ER*α* pathway as well as that of primary urothelium line predominantly via the ER*β* pathway [50].

Immunohistochemistry has detected ER*α* protein only in a small subset (e.g., 1–5%) of bladder cancer specimens [43, 52–54] (Table 1). Of note, in a study using a quantitative polymerase chain reaction (PCR) method,* ERα* gene was found to be positive in all the 10 tumors examined, which was even stronger (2.77-fold) than in matched normal tissues [50]. Our immunohistochemical analyses showed higher positive rates in bladder (27% [40]) and UUT (18% [46]) tumors, compared with those in other studies described above. In contrast to the findings in PCR analysis [50], elevated levels of ER*α* protein expression were detected in nonneoplastic urothelium, compared with urothelial cancer [40, 46, 53]. At least two of the immunohistochemical studies also demonstrated that ER*α* expression was downregulated in higher grade or stage tumors [40, 53]. However, no studies have identified the prognostic values of ER*α* in patients with urothelial tumor.

ER*β* protein expression was reported to be positive in 22–76% of urothelial tumors, which was significantly lower than the positive rates in nonneoplastic urothelial tissues, in some of the studies [30, 40, 41, 46, 52, 55, 56] (Table 1). More recently, Tan et al. [54] demonstrated that all the 410 bladder tumors examined were immunoreactive for ER*β*. There was significant upregulation [40, 44, 52, 55] or downregulation [56] of ER*β* expression seen in higher grade or more invasive tumors. Elevated ER*β* expression in bladder cancers was also found to correlate with higher risks of tumor recurrence and/or progression [30, 40, 57], and ER*β* positivity was an independent predictor of tumor progression [30]. Conversely, a strong association between ER*β* overexpression and favorable prognosis was demonstrated [44, 54].

## 4. Glucocorticoids and GR

The relationship between glucocorticoids and urothelial tumorigenesis is debatable. A population-based case-control study showed that prolonged oral glucocorticoid use was at an increased risk of developing bladder cancer [58], presumably due to immunosuppression. In contrast, our preclinical studies have revealed that glucocorticoids directly mediate GR activity in bladder cancer cells and that GR functions as a tumor suppressor [59, 60]. Natural or synthetic glucocorticoids, such as corticosterone, prednisone, and dexamethasone, strongly inhibited bladder cancer cell invasion and metastasis via inactivating nuclear factor- (NF-) *κ*B. However, treatment with dexamethasone resulted in an increase in bladder cancer cell viability and a decrease in apoptosis particularly that was induced by a cytotoxic agent, cisplatin, suggesting induction of chemoresistance by glucocorticoids. It is thus likely that GR signals, apart from glucocorticoid-induced immunosuppression, have dual roles in bladder cancer: suppression of tumor progression versus induction of cell proliferation. It should also be mentioned that the action of glucocorticoids is often complex and is generally dependent on a balance of transactivation and transrepression of GR that involve therapeutic effects of glucocorticoids and adverse effects associated with glucocorticoid therapy, respectively. Recently, we found that compound A, a plant derivative known to function as a GR agonist as well as an AR antagonist, induced only GR transrepression in bladder cancer cells and more efficiently inhibited tumor growth than dexamethasone or an antiandrogen flutamide [61].

Our immunohistochemical studies in bladder [62] and UUT [46] tumors showed that most of nonneoplastic urothelial tissues expressed the GR, which was downregulated in urothelial neoplasms (Table 1). GR expression was also significantly reduced in high-grade or muscle-invasive bladder tumors, compared with low-grade or non-muscle-invasive tumors [62]. However, this was not seen in UUT tumors [46]. In addition, loss of GR expression was found to correlate with recurrence of non-muscle-invasive bladder tumors and progression of muscle-invasive bladder tumors in univariate analyses [62]. Multivariate analysis identified low GR expression as a predictor for recurrence of non-muscle-invasive bladder tumors (hazard ratio (HR) = 2.252; *P* = 0.034) and progression of muscle-invasive bladder tumors (HR = 3.690; *P* = 0.077). However, the levels of GR expression were not significantly associated with the prognosis of the patients with UUT tumor in our study [46].

## 5. Progestogens and PR

A case-control study demonstrated significant decreases in bladder cancer incidence in multiparous women or women with oral contraceptive use [63]. In a study using a transgenic model for bladder cancer, multiparous female mice developed significantly smaller tumors than nulliparous females [64]. These observations imply benefits of not only estrogens but also progestogens for preventing the development of urothelial tumors.

Hormone-binding assay showed that 1 of 3 noninvasive and 3 of 3 advanced urothelial tumors were positive for PR [65]. An immunohistochemical study also demonstrated PR expression in the urothelium in 18 of 20 bladders from male children aged 1–12 [66]. Nonetheless, two subsequent immunohistochemical studies in 198 [53] and 410 [54] bladder cancer specimens failed to detect PR signals (Table 1). In another study of bladder tumors, the positive rates of PR were 2% and 4% in nonneoplastic urothelium and carcinoma tissues, respectively [43]. We recently showed that 13% of nonneoplastic urothelial tissues from the UUT and 16% of UUT tumors were immunoreactive for PR [46]. There was no significant difference in PR expression between low-grade versus high-grade or non-muscle-invasive versus muscle-invasive UUT tumors. Interestingly, in our study, PR positivity in pT3 or pT4 UUT tumors was strongly associated with disease-specific mortality.

## 6. Vitamin D and VDR

Low serum levels of vitamin D have been implicated in the risk of bladder cancer [67].* VDR* gene polymorphism resulting in reduction of receptor activity has also been correlated with higher incidence of bladder cancer [68]. Furthermore, vitamin D was shown to prevent bladder tumorigenesis in rats treated with a carcinogen N-methylnitrosourea as well as to inhibit cell growth of VDR-positive bladder cancer lines [69]. Thus, VDR signals appear to play a protective role in bladder tumor outgrowth.

VDR was found positive immunohistochemically in 86–100% of bladder tumors [70, 71] (Table 1). In contrast to the above findings, however, upregulation of VDR expression was seen in high-grade and muscle-invasive tumors, compared with low-grade and non-muscle-invasive tumors, respectively, in one of the studies [70]. Strong VDR expression was significantly associated with lower progression-free survival and cancer-specific survival rates.

## 7. Retinoic Acids and Retinoid Receptors

The preventive effects of retinoic acids, including vitamin A and its derivatives, on bladder cancer development have been assessed. A recent meta-analysis involving 25 studies demonstrated a significant inverse association between dietary intake of vitamin A/retinol and bladder cancer risk [72]. Preclinical studies also showed that retinoids inhibited bladder carcinogenesis in animals treated with BBN [73] and cell proliferation of bladder cancer lines [74].

In a study using a PCR-based method, all of the nonneoplastic bladders were found to express the retinoid receptors [75]. However, some of muscle-invasive bladder cancers lost RAR*α* (60%), RAR*γ* (20%), and RXR*α* (40%), while they were positive in all non-muscle-invasive tumors. RAR*β*2 was positive in 50% of non-muscle-invasive tumors and 40% of muscle-invasive tumors. In addition, methylated RAR*β* was frequently found in bladder cancer tissues and urine samples from bladder cancer patients [76–78], suggesting its utility as a urine marker. Specifically, the sensitivity of RAR*β* for tumor detection was higher than that of urine cytology (68% versus 46% for all cases; 67% versus 11% for grade 1 tumors) [77].

## 8. PPARs

There has been a link between the use of pioglitazone, a PPAR agonist prescribed as a hypoglycemic drug, and bladder cancer risk [79]. Indeed, treatment with a PPAR*γ* agonist rosiglitazone or PPAR*γ* overexpression resulted in significant increases in bladder cancer cell migration and invasion [80]. Earlier studies conversely showed that PPAR*γ* agonists inhibited bladder cancer cell growth [81, 82]. Of note, there appear to be multiple mechanisms for inducing antitumor effects of PPAR*γ* agonists, some of which are independent of PPAR*γ* signals [83]. Additionally,* in situ* hybridization showed that* PPARγ* gene was often amplified in bladder cancer specimens [80, 81].

## 9. Orphan Nuclear Receptors

Okegawa et al. recently demonstrated up- or downregulation of a variety of orphan nuclear receptor genes in bladder cancer tissues, compared with paired normal bladders [84]. Of these receptors, hepatocyte nuclear factor 4*γ* (HNF4G) was most frequently elevated in tumors and its overexpression promoted tumor growth in both* in vitro* and* in vivo* [84]. Nurr1 was also often overexpressed in bladder cancers [84, 85], which correlated with the promotion of bladder cancer cell migration [85]. Immunohistochemistry of Nurr1 in bladder cancer specimens showed significant increases in its expression levels in higher grade/stage tumors [85] (Table 1). Moreover, high cytoplasmic Nurr1 expression, but not total expression, was an independent prognosticator of cancer-specific mortality (HR = 4.894; *P* < 0.001) [85]. Similarly, Nur77 was overexpressed especially in muscle-invasive bladder cancers [84, 86]. However, Nur77 activation correlated with retardation of bladder tumor growth in cell line and animal models [86, 87].

## 10. Molecules Regulated by Steroid Hormone Receptor Signaling

Increasing evidence suggests the involvement of upstream pathways as well as downstream targets of steroid hormone receptor-mediated signals in the development and progression of urothelial cancer.  Table 2 summarizes such molecules directly or indirectly regulated by AR and/or ER signals. The following are key molecules that androgens/estrogens have been shown to up- or downregulate via the AR/ER pathways in bladder cancer cells.

### 10.1. CD24

AR signals activate CD24, a glycoprotein and a cell adhesion molecule, in bladder cancer cells [88]. In animal models, CD24 overexpression and knockdown resulted in stimulation and inhibition, respectively, of the development of primary bladder cancer and its metastasis [88, 89]. Immunohistochemical analyses in bladder cancer specimens [89–91] have revealed that CD24 is expressed exclusively in tumor cells, but not in surrounding stromal cells. These studies also showed higher levels of CD24 expression in grade 2-3 tumors (74%) than in grade 1 tumors (28%; *P* < 0.001) [90], in ≥pT3 tumors than in ≤pT2 tumors (*P* = 0.036) [91], or in metastatic tumors (93%) than in primary tumors (75%; *P* = 0.006) [89]. Furthermore, elevated CD24 expression was associated with recurrence of non-muscle-invasive tumors (*P* < 0.001 for all cases or grades 2-3; *P* = 0.042 for grade 1) [90] or cancer-specific mortality in patients with muscle-invasive tumor (*P* < 0.001) [91] in univariate settings. However, CD24 was not an independent prognosticator for muscle-invasive bladder cancers (HR = 1.12; *P* = 0.84) [91].

### 10.2. *β*-Catenin

AR signals activate Wnt/*β*-catenin signaling in bladder cancer cells [92, 93]. *β*-Catenin, as a key component of the Wnt signaling pathway, is a multifunctional protein and is known to activate target genes, such as the protooncogene* c-myc*, the cell cycle activator* cyclin D1*, and the* epidermal growth factor receptor* (*EGFR*). Using an animal model for bladder cancer, *β*-catenin was shown to induce tumorigenesis, and androgen-mediated AR signals appeared to synergize with *β*-catenin [93]. There are conflicting data as to the correlation of *β*-catenin staining in bladder cancer specimens with tumor aggressiveness. Consistent with the findings in other studies [94, 95], we observed downregulation of membranous *β*-catenin expression in bladder cancer, compared with nonneoplastic urothelium [92]. In addition, loss or reduced expression of membranous *β*-catenin, as well as nuclear accumulation of *β*-catenin as a hallmark of Wnt/*β*-catenin activation, correlated with higher tumor grade, more advanced tumor stage, and/or worse patient outcomes [42, 92, 94, 96]. Coexpression of nuclear *β*-catenin and AR in bladder cancer cells was also noted [42, 92].

### 10.3. Slug

Androgens were shown to upregulate Slug expression in bladder cancer cells, which could subsequently induce epithelial-to-mesenchymal transition through the activation of Wnt/*β*-catenin signaling [42]. Slug expression was significantly upregulated in high-stage bladder cancers (e.g., non-muscle-invasive 27% versus muscle-invasive 77%, *P* = 0.023 [42]; lymph node-negative 58% versus lymph node-positive 89%, *P* = 0.012 [97]; non-muscle-invasive 23% versus muscle-invasive 77%, *P* = 0.04 [98]), whereas there were no statistically significant differences in Slug expression between low-grade and high-grade tumors in these 3 studies. Prognostic significance of Slug expression in bladder tumors was not seen or was not assessed in these studies.

### 10.4. EGFR/ERBB2

Activation of the EGFR family, such as EGFR and ERBB2, is known to involve bladder tumorigenesis and cancer progression. Accordingly, the efficacy of targeted therapy directed at EGFR signals has been assessed in bladder cancer [99–104]. We demonstrated that androgen upregulated the expression of EGFR and ERBB2 as well as the levels of phosphorylation of their downstream proteins AKT and extracellular signal-regulated kinase- (ERK-) 1/2 via the AR pathway in bladder cancer cells [31]. EGF could also induce bladder cancer cell proliferation via modulating AR signals [32]. Alterations of the EGFR family, such as protein overexpression and gene amplification or mutation, have been extensively studied in bladder cancer specimens, providing mixed results regarding their prognostic values [104–109]. For instance, some studies suggested that ERBB2 overexpression was a poor prognostic factor, while others did not. Nonetheless, ERBB2 was found to be overexpressed in muscle-invasive bladder cancers in most of the studies.

### 10.5. UGT1A

UGT1A, a major phase II drug metabolism enzyme, plays a critical role in detoxifying bladder carcinogens. In a normal urothelial cell line SVHUC as well as in normal mouse bladders, androgens/estrogens decreased/increased the expression levels of UGT1A and its subtypes via the AR/ER*β* pathways, respectively [28, 110]. An initial immunohistochemical study showed that 6 of 19 bladder tumors lost UGT1A, while benign tissues consistently expressed it [111]. Our immunohistochemical staining subsequently showed reduced expression of UGT1A in 145 urothelial neoplasms, compared with paired nonneoplastic urothelial tissues, as well as inverse correlations between UGT1A levels and tumor grade or pT stage [110]. Decreased UGT1A expression was also strongly associated with the progression of high-grade non-muscle-invasive tumors (*P* = 0.038) or worse cancer-specific survival in patients with muscle-invasive tumor (*P* = 0.016), and the latter was an independent prognosticator (HR = 3.413; *P* = 0.010) [110]. In addition, the expression of UGT1A was positively and negatively correlated with the levels of ER*α* and ER*β*, respectively.

### 10.6. ELK1

ELK1, a member of the ETS-domain family of transcription factors, is known to involve cell proliferation, cell cycle control, and apoptosis via regulating the expression of a variety of genes, including* c-fos* protooncogene. We recently demonstrated that androgens activated ELK1 in bladder cancer cells and promoted the proliferation of only ELK1-positive cells and the migration/invasion of both ELK1-positive and ELK1-negative cells [33]. Androgens also failed to significantly induce AR transcriptional activity in ELK1 knockdown bladder cancer cells. Our immunohistochemical staining showed significant increases in the expression of ELK1 and phospho-ELK1 (an activated form of ELK1) in bladder tumors, compared with nonneoplastic urothelial tissues [33]. The expression of ELK1/phospho-ELK1 versus AR was significantly correlated. While there were no significant correlations between the levels of ELK1 or phospho-ELK1 and tumor grades or stages, phospho-ELK1 positivity precisely predicted the recurrence of non-muscle-invasive tumors in a univariate setting (*P* = 0.043) as well as a worse outcome of muscle-invasive tumors in both univariate (*P* = 0.045 for disease progression; *P* = 0.008 for cancer-specific mortality) and multivariate (HR = 2.693; *P* = 0.021 for cancer-specific mortality) settings. Subsequent immunohistochemistry in bladder cancer specimens from patients who received neoadjuvant chemotherapy revealed that phospho-ELK1 positivity strongly correlated with chemoresistance [112]. Indeed, ELK1 inactivation resulted in enhancement of the cytotoxic activity of cisplatin in bladder cancer cells [112].

### 10.7. GATA3

GATA3, a member of the GATA family of zinc-finger transcription factors, has recently been recognized as a urothelial marker and its immunohistochemistry has therefore been widely used in diagnostic surgical pathology [113–115]. Using SVHUC cells with carcinogen challenge, we demonstrated that GATA3 strongly prevented neoplastic transformation of urothelial cells [29]. GATA3 knockdown in SVHUC exposed to the chemical carcinogen resulted in downregulation of the molecules that play a protective role in bladder tumorigenesis, such as UGT1A, PTEN, p53, and p21, and upregulation of oncogenic genes, such as c-myc, cyclins D1/D3/E, and FGFR3. Additionally, similar to the findings in UGT1A described above, androgens/estrogens down/upregulated GATA3 expression in nonneoplastic urothelial cells via the AR/ER*β* pathways, respectively [29]. GATA3 knockdown in bladder cancer lines also resulted in promotion of cell invasion and migration as well as induction of the expression of their related molecules, such as MMP-2 and MMP-9 [116], while androgens did not significantly change the levels of GATA3 expression in these cells [29]. Our immunohistochemical data showed that GATA3 was positive in 98% of nonneoplastic urothelial tissues versus 86% of urothelial neoplasms as well as in 98% of low-grade and/or non-muscle-invasive tumors versus 72–80% of high-grade and/or muscle-invasive tumors [117]. In tumors, there were strong correlations between GATA3 expression versus AR overexpression, ER*α* overexpression, or loss of ER*β* expression. We also demonstrated that patients with GATA3-positive muscle-invasive tumor had a significantly higher risk of disease progression in a univariate setting (*P* = 0.048) and, in this subgroup, strong GATA3 expression was correlated with tumor progression (HR = 2.435; *P* = 0.052) or cancer-specific survival (HR = 3.673; *P* = 0.040) in a multivariate setting [117].

### 10.8. Inositol Polyphosphate 4-Phosphatase Type II (INPP4B)

INPP4B has been recognized as a tumor suppressor of several types of malignancies, such as breast and prostate cancers, but its role in bladder cancer remained unclear. In bladder cancer cells, estrogens were shown to upregulate INPP4B via the ER*α* pathway, resulting in inhibition of AKT activity and cell growth [51]. Chromatin immunoprecipitation assay further revealed that ER*α* could bind to a putative estrogen response element region of the INPP4B promoter in bladder cancer cells. Immunohistochemistry showed that INPP4B was positive in 62% of bladder tumors, which was significantly lower than in benign urothelial tissues (87%; *P* < 0.001) [51]. Similarly, 75% of low-grade versus 53% of high-grade tumors (*P* = 0.016) as well as 74% of non-muscle-invasive versus 44% of muscle-invasive tumors (*P* < 0.001) were INPP4B-positive. There was also a positive correlation between INPP4B expression and ER*α* expression. However, no prognostic significance of INPP4B expression in bladder tumors has been demonstrated.

## 11. Conclusion

Mounting evidence suggests that steroid hormone receptor-mediated signals play a critical role in urothelial tumorigenesis and cancer progression. Various molecules, as downstream targets, have also been shown to be modulated by these signals. Immunohistochemical studies in surgical specimens have identified significant differences in the expression levels of several steroid hormone receptors and their related proteins between nonneoplastic urothelium versus urothelial tumor and between low-grade/non-muscle-invasive versus high-grade/muscle-invasive urothelial tumors. More importantly, although the underlying mechanisms of how steroid hormone receptors and related signals regulate urothelial tumor outgrowth remain far from being fully understood, the available data support that these can serve as biomarkers of urothelial tumors, especially their prognosticators. Further investigation of steroid hormone receptors as well as other molecules directly or indirectly regulated by steroid hormones may help develop not only better strategies for the management of urothelial tumors but also more reliable biomarkers.

## Figures and Tables

**Table 1:** Immunohistochemical studies for the expression of steroid hormone receptors in urothelial carcinoma specimens.

Author, year [reference]	Receptor	*N*	Location	Nontumor (nonneoplastic urothelium) versus tumor			Tumor grade			Tumor stage			Prognostic significance (*P* value)
				Non-Tumor	Tumor	*P* value	LG	HG	*P* value	NMI	MI	*P* value	
Boorjian et al., 2004 [36]	AR	49	Bladder	86%	53%	0.001^*∗*^	89%	49%	0.055^*∗*^	75%	21%	0.002^*∗*^	NA
Boorjian et al., 2009 [25]	AR	55	Bladder	NA	44%	0.06	NA	NA	NA	59%	33%	0.095^*∗*^	NA
Kauffman et al., 2011 [37]	AR	59	Bladder	84%	Roughly half	<0.001	NA	NA	NA	NA	NA	0.028 (NMI > MI)	NS
Mir et al., 2011 [38]	AR	472	Bladder	NA	13%	NA	12%	13%	0.83	9%	15%	0.058	NS
Rau et al., 2011 [39]	AR	93	UUT	NA	NA	NA	NA	NA	0.074 (LG > HG)	NA	NA	0.001 (stage II > I)	0.568
Tuygun et al., 2011 [30]	AR	139	Bladder	0% (M)	51%	<0.001^*∗*^	64%	37%	0.002^*∗*^	60%	21%	<0.001^*∗*^	0.095 (RFS) 0.110 (PFS)
Miyamoto et al., 2012 [40]	AR	188	Bladder	80%	42%	<0.001	55%	36%	0.023	51%	33%	0.018	0.071 (PFS/MI)
Shyr et al., 2013 [41]	AR	83	UUT	NA	55%	NA	69%	47%	0.070^*∗*^	63%	44%	0.114^*∗*^	NA
Jing et al., 2014 [42]	AR	58	Bladder	NA	53%	NA	55%	50%	0.724	49%	69%	0.195	NA
Mashhadi et al., 2014 [43]	AR	120	Bladder	0%	22%	<0.001	NA	NA	<0.001	NA	NA	<0.001	0.02
Nam et al., 2014 [44]	AR	169	Bladder	NA	37%	NA	39%	33%	0.269	43% (Ta) 30% (T1)	NA	0.048 (Ta vs T1)	0.001 (RFS) 0.288 (PFS)
Williams et al., 2015 [45]	AR	297	Bladder	NA	25%	NA	NA	NA	NA	33% (Ta/Tis)	19% (T1–3)	0.010^*∗*^	NA
Williams et al., 2015 [45]	AR	43	UUT	NA	16%	NA	NA	NA	NA	NA	NA	NA	NA
Kashiwagi et al., 2015 [46], and unpublished data	AR	99	UUT	58%	20%	<0.001	33%	18%	0.177	14%	24%	0.301	NS

Shen et al., 2006 [52]	ER*α*	224	Bladder	NA	1%	NA	NA	NA	NA	NA	NA	NA	NA
Bolenz et al., 2009 [53]	ER*α*	198	Bladder	NA	5%	0.06	NA	NA	NA	NA	NA	0.004 (OC > non-OC)	NS
Miyamoto et al., 2012 [40]	ER*α*	188	Bladder	50%	27%	<0.001	38%	23%	0.048	35%	19%	0.014	NS
Mashhadi et al., 2014 [43]	ER*α*	120	Bladder	2%	3%	0.67	NA	NA	NA	NA	NA	NA	NA
Tan et al., 2015 [54]	ER*α*	410	Bladder	NA	4%	NA	NA	NS	NA	NA	NS	NA	NA
Kashiwagi et al., 2015 [46], and unpublished data	ER*α*	99	UUT	40%	18%	0.001	27%	17%	0.465	11%	23%	0.183	NS

Croft et al., 2005 [55]	ER*β*	92	Bladder	NA	11–22%^*∗*^	NA	6–12%^*∗*^ (G1-2)	17–33%^*∗*^ (G3)	0.021–0.177^*∗*,*∗∗*^	5–9%^*∗*^ (Ta)	16–33%^*∗*^ (≥T1)	0.010–0.098^*∗*,*∗∗*^	NA
Shen et al., 2006 [52]	ER*β*	224	Bladder	NA	63%	NA	58% (G1-2)	70% (G3)	0.085	54%	80%	<0.001	NA
Kontos et al., 2010 [56]	ER*β*	111	Bladder	93%	76%	0.041^*∗*^	95% (G1-2)	56% (G3)	<0.001^*∗*^	83% (T1)	54%	0.011^*∗*^	NA
Tuygun et al., 2011 [30]	ER*β*	139	Bladder	0% (M) 36% (F)	27–30%^*∗∗*^	<0.001^*∗*^	22–26%^*∗∗*^	31–34%^*∗∗*^	0.44–0.59^*∗∗*^	24–26%^*∗∗*^	36–42%^*∗∗*^	0.16–0.24^*∗∗*^	0.114 (RFS) 0.025 (PFS)
Miyamoto et al., 2012 [40]	ER*β*	188	Bladder	89%	49%	<0.001	29%	58%	<0.001	34%	67%	<0.001	0.007 (PFS/LG) ≤0.001 (PFS) 0.007 (CSS/MI)
Kauffman et al., 2013 [57]	ER*β*	72	Bladder	NA	NA	<0.001 (N < T)	NA	NA	NA	NA	NA	NS	0.030 (RFS) 0.0018 (CSS)
Shyr et al., 2013 [41]	ER*β*	83	UUT	NA	43%	NA	44%	43%	1.000^*∗*^	51%	47%	0.815^*∗*^	NA
Nam et al., 2014 [44]	ER*β*	169	Bladder	NA	31%	NA	27%	41%	0.043	22% (Ta) 42% (T1)	NA	0.004 (Ta vs T1)	0.004 (RFS) 0.014 (PFS)
Tan et al., 2015 [54]	ER*β*	410	Bladder	NA	100%	NA	100%	100%	NS	100%	100%	NS	0.055/0.087 (CSS)
Kashiwagi et al., 2015 [46], and unpublished data	ER*β*	99	UUT	85%	63%	0.001	73%	61%	0.402	65%	63%	1.000	NS

Ishiguro et al., 2014 [62]	GR	149	Bladder	96%	87%	0.026	96%	81%	0.011	96%	74%	<0.001	0.025 (RFS/NMI) 0.030 (PFS/MI) 0.067 (CSS/MI)
Kashiwagi et al., 2015 [46], and unpublished data	GR	99	UUT	84%	63%	0.001	53%	64%	0.563	62%	63%	1.000	NS

Bolenz et al., 2009 [53]	PR	198	Bladder	NA	0%	NA	NA	NA	NA	NA	NA	NA	NA
Mashhadi et al., 2014 [43]	PR	120	Bladder	2%	4%	0.48	NA	NA	NA	NA	NA	NA	NA
Tan et al., 2015 [54]	PR	410	Bladder	NA	0%	NA	0%	0%	NS	0%	0%	NS	NA
Kashiwagi et al., 2015 [46], and unpublished data	PR	99	UUT	13%	16%	0.487	7%	18%	0.453	14%	18%	0.779	0.041 (CSS/pT3-4)

Hermann and Andersen, 1997 [70]	VDR	26	Bladder	NA	100%	NA	100%	100%	0.043^*∗*^ (LG < HG)	100%	100%	0.051^*∗*^ (NMI < MI)	NA
Sahin et al., 2005 [71]	VDR	105	Bladder	67%	86%	0.02	81% (G1)	91% (G2-3)	NS	85% (Ta) 87% (T1)	NA	NS	0.001 (PFS)

Inamoto et al., 2010 [85]	Nurr1	145	Bladder	0% (high)	100% 65% (high)	NA	35% (high; G1-2)	92% (high; G3)	<0.001	47% (high)	92% (high; including T1b)	<0.001	<0.001 (RFS)^*∗∗∗*^ <0.001 (CSS)^*∗∗∗*^

AR: androgen receptor; ER: estrogen receptor; GR: glucocorticoid receptor; PR: progesterone receptor; VDR: vitamin D receptor; UUT: upper urinary tract; NA: not analyzed; M: males; F: females; LG: low-grade; HG: high-grade; NMI: non-muscle-invasive; MI: muscle-invasive; OC: organ-confined; RFS: recurrence-free survival; PFS: progression-free survival; CSS: cancer-specific survival; NS: not significant

^*∗*^We calculated the two-tailed *P* values using Fisher's exact test.

^*∗∗*^Two criteria.

^*∗∗∗*^Cytoplasmic expression.

**Table 2:** Molecules regulated by sex hormone receptor signaling in urothelial carcinoma.

	Associated receptor signaling	Effect on urothelial carcinogenesis and/or cancer progression	Hormone effect	Reference
CD24	AR	Stimulation	Upregulation	[88]
*β*-catenin	AR	Stimulation	Upregulation	[92, 93]
Slug	AR	Stimulation	Upregulation	[42]
EGFR	AR	Stimulation	Upregulation	[31]
ERBB2	AR	Stimulation	Upregulation	[31]
Akt	AR/ER*α*	Stimulation	Upregulation/downregulation	[31, 51]
ERK1/2	AR	Stimulation	Upregulation	[31]
Cyclin D1	AR	Stimulation	Upregulation	[26]
Cyclin D3	AR	Stimulation	Upregulation	[29]
Cyclin E	AR	Stimulation	Upregulation	[29]
FGFR3	AR	Stimulation	Upregulation	[29]
UGT1A	AR/ER*β*	Inhibition	Downregulation/upregulation (SVHUC)	[28, 110]
p53	AR	Inhibition	Downregulation	[29, 118]
p21	AR	Inhibition	Downregulation	[29, 118]
PTEN	AR	Inhibition	Downregulation	[29]
c-myc	AR	Stimulation	Upregulation	[29]
Bcl-xL	AR	Stimulation	Upregulation	[26]
MMP-9	AR	Stimulation	Upregulation	[26]
ELK1	AR	Stimulation	Up-regulation	[33]
GATA3	AR/ER*β*	Inhibition	Downregulation/upregulation (SVHUC)	[29]
INPP4B	ER*α*	Inhibition	Upregulation	[51]

AR: androgen receptor; ER: estrogen receptor.
